# Determination of magnetic anisotropy constants and domain wall pinning energy of Fe/MgO(001) ultrathin film by anisotropic magnetoresistance

**DOI:** 10.1038/srep14114

**Published:** 2015-09-15

**Authors:** Bo Hu, Wei He, Jun Ye, Jin Tang, Yong-Sheng Zhang, Syed Sheraz Ahmad, Xiang-Qun Zhang, Zhao-Hua Cheng

**Affiliations:** 1 State Key Laboratory of Magnetism and Beijing National Laboratory for Condensed Matter Physics, Institute of Physics, Chinese Academy of Sciences, Beijing 100190, China

## Abstract

It is challenging to determine domain wall pinning energy and magnetic anisotropy since both coherent rotation and domain wall displacement coexist during magnetization switching process. Here, angular dependence anisotropic magnetoresistance (AMR) measurements at different magnetic fields were employed to determine magnetic anisotropy constants and domain wall pinning energy of Fe/MgO(001) ultrathin film. The AMR curves at magnetic fields which are high enough to ensure the coherent rotation of magnetization indicate a smooth behavior without hysteresis between clockwise (CW) and counter-clockwise (CCW) rotations. By analyzing magnetic torque, the magnetic anisotropy constants can be obtained. On the other hand, the AMR curves at low fields show abrupt transitions with hysteresis between CW and CCW rotations, suggesting the presence of multi-domain structures. The domain wall pinning energy can be obtained by analyzing different behaviors of AMR. Our work suggests that AMR measurements can be employed to figure out precisely the contributions of magnetic anisotropy and domain wall pinning energy, which is still a critical issue for spintronics.

The magnetic properties of Fe film epitaxially grown on MgO(001) substrate have attracted much attention since the discovery of a very high tunneling magnetoresistance ratio in Fe/MgO/Fe magnetic tunneling junction^1^,^2^,^3^. It is well known that the magnetization switching process of Fe/MgO(001) is crucial for spintronic applications^4^. Although Fe(001) film usually exhibits an intrinsically in-plane four-fold magnetocrystalline anisotropy, an additional uniaxial magnetic anisotropy (UMA)^5^ is always superimposed on the magnetocrystalline anisotropy owing to the surface steps of substrates^6^, oblique deposition^7^ or dangling bonds^8^. Depending upon the orientation of the applied field and the strength of UMA, the UMA profoundly affects the magnetization switching process, leading to “one-jump”, “two-jump” or other types of magnetic hysteresis loop^9^,^10^. When the ratio of the four-fold magnetic anisotropy constant *K*_1_ and UMA constant *K*_*U*_, *K*_*U*_*/K*_*1*_ < *1*, two-jump magnetization switching process will appear in the hysteresis loops of Fe(001)/MgO(001) film^9^. The two-jump magnetization switching process can be explained by competition of the 90° domain wall pinning energy and magnetic anisotropy energy^11^,^12^.

A fundamental understanding of the evolving magnetic anisotropy and domain wall pinning energy remains elusive and is still a critically technological issue because they determine the magnetization switching process and the dynamic response on nanoscale^13^,^14^. The domain wall pinning energy and magnetic anisotropy can be separately investigated by various experimental methods^9^,^15^,^16^. Unfortunately, it is challenging to investigate the domain wall pinning energy and magnetic anisotropy of Fe/MgO(001) film simultaneously by a single method since both coherent rotation and domain wall displacement coexist during magnetization switching process.

In this paper, the angular dependence anisotropic magnetoresistance (AMR) measurement was introduced to investigate magnetization switching process in Fe(001) film on MgO(001) substrate. By carefully analyzing angular dependence AMR at high fields and low fields, the magnitudes of additional UMA and four-fold magnetic anisotropy constants as well as the values of domain wall pinning energy can be obtained, respectively. The contributions of magnetic anisotropy and domain wall pinning energy of Fe/MgO(001) film can be probed precisely by AMR measurements in our work.

## Results

**Figure 1:**
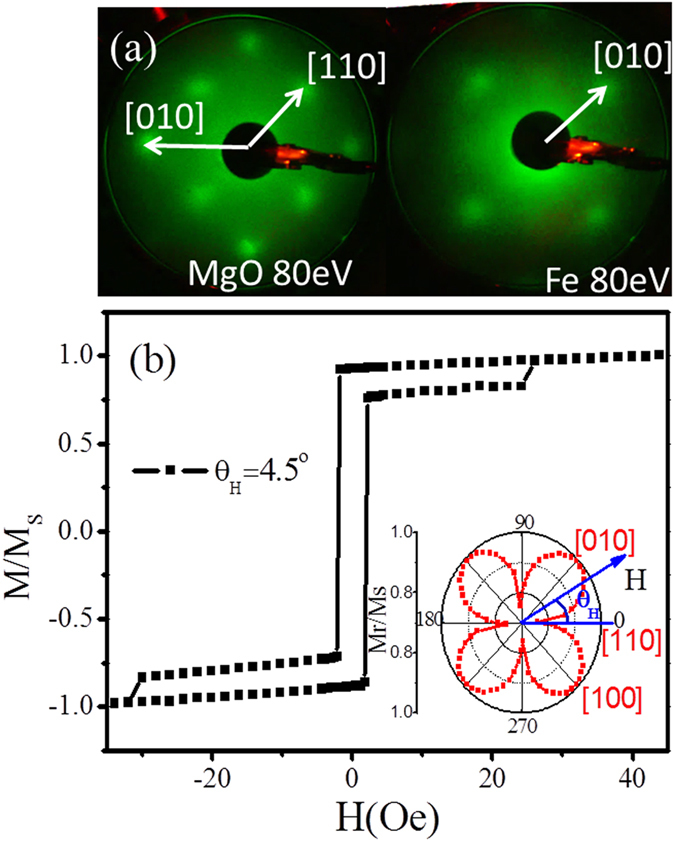
The typical LEED patterns of the substrate and sample, and MOKE measurement. (**a**) The typical LEED patterns of MgO(001) substrate and Fe(001) films, (**b**) The hysteresis loop of Fe/MgO(001) measured at *θ*_*H*_ = 4.5° near hard axis by MOKE measurement. The inset indicates the angular dependence of *M*_*r*_*/M*_*S*_ and direction of Fe film hard and easy axes.

As shown in the left panel of Fig. 1(a), the typical low-energy electron diffraction (LEED) pattern indicates a clean MgO(001) substrate surface. Due to the relatively small lattice mismatch of Fe(001)||MgO(001) and Fe[100]||MgO[110] (3.8%)^17^, the Fe(001) layers grow epitaxially with *bcc* lattice structure on the MgO(001) surface. A tetragonal distortion results in epitaxial relationship between Fe layer and MgO substrate in 45° in-plane rotation^7^,^18^. The good quality of Fe film is also verified by *in-situ* LEED pattern (right panel of Fig. 1(a)). The hysteresis loop of Fe/Mg(001) film characterized by longitudinal magneto-optical Kerr effect (MOKE) exhibits two-jump magnetization switching process, which can also be observed in other literature in the case of *K*_*U*_*/K*_*1*_ < 1^9^. Moreover, angular dependence *M*_*r*_*/M*_*S*_ as shown in the inset of Fig. 1(b) indicates four-fold magnetic anisotropy of film. From the LEED pattern and in-plane MOKE analysis, Fe[110] and [100] axes can be confirmed as shown in Fig. 1(a).

**Figure 2:**
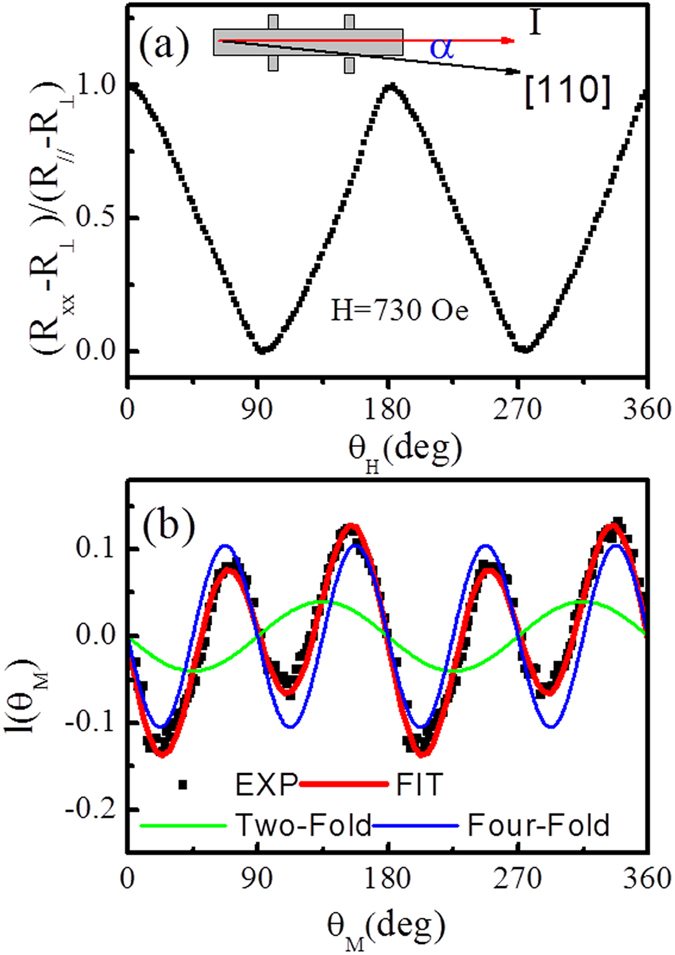
The in-plane AMR curve and the normalized magnetic torque curve. (**a**) The angular dependence AMR measurement at high field of 730 *Oe*, (**b**) The experimental and simulated normalized magnetic torque curves.

The two-jump magnetization switching process is related to the *K*_*1*_ and *K*_*U*_. In order to figure out those parameters, the angular dependence AMR at high field of 730 *Oe* was measured as shown in Fig. 2(a). The AMR can be expressed as Eq. (1)^16^,^19^,^20^,^21^,^22^,^23^:


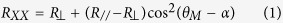


where *θ*_*M*_ and *α* are angles of magnetic moment *M* and current *I* measured from the Fe[110] direction. The current was applied at an angle *α* = 6.3° with respect to Fe [110] for AMR measurements, which will be discussed in details later. The maximum value *R*_//_ and minimum value 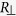 are corresponding to AMR when *H* is parallel and perpendicular to the direction of current, respectively. By changing the direction of applied field, the *M* follows the orientation of external field and the values of AMR show a periodically oscillated behavior. However, due to the magnetic anisotropy, *M* is no longer kept along with the external field *H* during rotation^22^. The AMR value is related to *θ*_*M*_ on basis of Eq. (1). The normalized magnetic torque 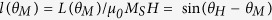 can be obtained.

Since the applied field of 730 *Oe* is large enough to guarantee a single domain rotation in Fe/MgO(001) system, the total energy consisting of the magnetic anisotropy energy and Zeeman energy can be expressed as Eq. (2)^15^.


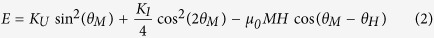


In equilibrium state 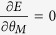, the normalized magnetic torque is:


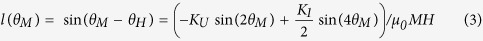


By fitting the magnetic torque curve by Eq. (3), which is shown in Fig. 2(b), *K*_1_ = 2.67 × 10^4^ *J/m*^*3*^ and *K*_*U*_ = 4.2 × 10^3^ *J/m*^*3*^ can be obtained.

It can be observed from Fig. 2(b) that the magnetic torque shows a superposition of two- and four-fold magnetic anisotropies from the UMA constant *K*_*U*_ and the four-fold magnetic anisotropy constant *K*_1_, respectively. The competition between *K*_*1*_ and *K*_*U*_ leads to a slight deviation of easy magnetization axis about 
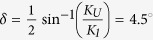
 from Fe[100] direction^9^.

**Figure 3:**
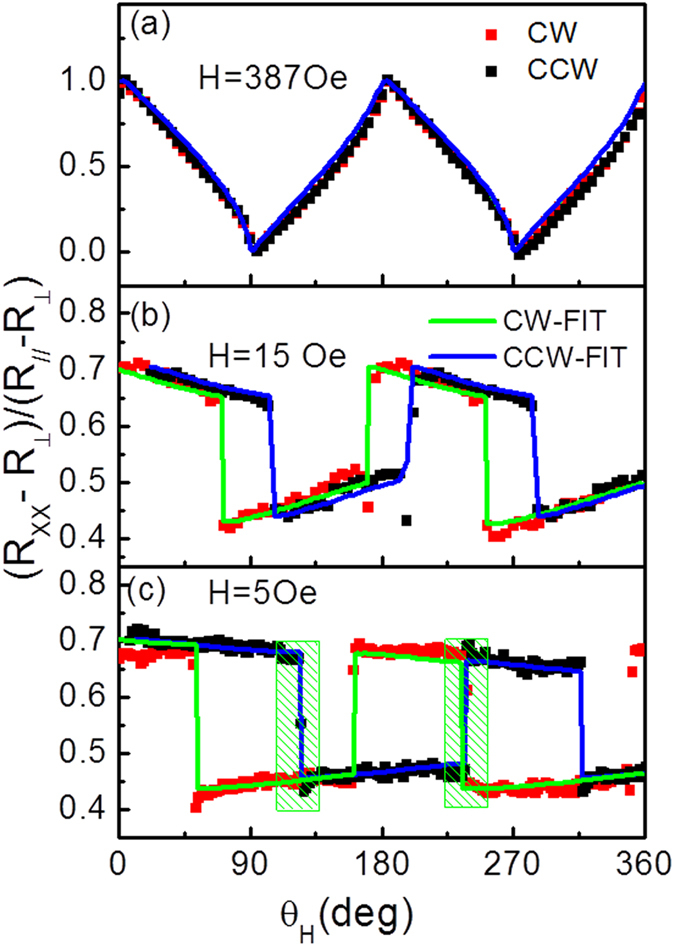
The experimental and fitting angular dependences of AMR data at different fields (**a**) 387 *Oe*, (**b**) 15 *Oe* and (**c**) 5 *Oe* with different rotation directions. The experimental data (red and black symbols) and the fitting data (green and blue solid line) are taken with field rotations in the clockwise (CW) and counter clockwise (CCW) directions.

**Figure 4:**
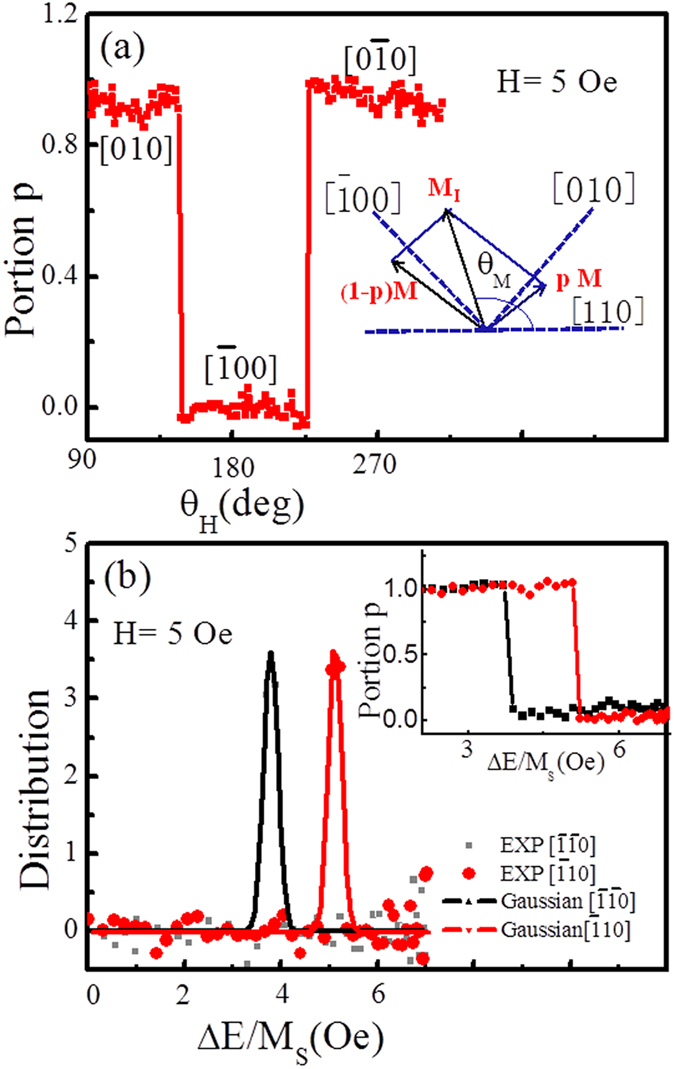
Probability of finding magnetic domains oriented along the [010] direction and the distributions of pinning fields. (**a**) The fraction *p* varying with magnetic flied direction and inset shows configuration of two magnetic domains for an intermediate state producing a resultant magnetization between two easy axes. (**b**) The distributions of pinning fields were obtained by derivative of *p* with respect to Δ*E/M*_*s*_. The relation between *p* and the pinning field Δ*E/M*_*s*_ for crossing two hard axes as shown in inset figure.

Figure 3 illustrates the angular dependence of AMR at different applied magnetic fields with clockwise (CW) and counterclockwise (CCW) rotations. The AMR curves at high magnetic field of 387 *Oe* shown in Fig. 3(a) indicate a smooth behavior without hysteresis between CW and CCW rotations, implying a coherent rotation of magnetization in this field. Similar with planar Hall effect in GaMnAs films^13^,^24^,^25^, the AMR curves at low fields show abrupt transitions at certain angles with hysteresis between CW and CCW rotations, suggesting the presence of multi-domain structures. In order to investigate the domain structures, we focus on the abrupt transition regions (shaded regions) at low field *H* = 5 *Oe* in Fig. 3(c). The current is applied at an angle of 6.3° with respect to the Fe[110] direction to distinguish two components of magnetization along the two easy axes, which makes AMR reach minimum and maximum values when *M* is along 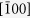 and [010] directions, respectively. The AMR at low field of 5 *Oe* (Fig. 3(c)) is taken due to its quite plateau between two abrupt transitions, indicating that 90° domain nucleation and propagation, as observed by Kerr microscope and discussed in ref. 24. Under this low field, the direction of *M* switches from the Fe[010] direction to Fe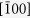 and Fe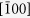 to 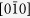. The magnetization in this transition region can be calculated from 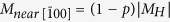, where *p* is the fraction of *M*_[010]_ as shown in inset of Fig. 4(a) 26. According to Eq. (1), when *M* is near Fe[010] and Fe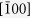, AMR is in the high resistance state *R*_*H*_ (Eq. (4)), and the low resistance state *R*_*L*_ (Eq. (5)), respectively.


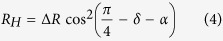



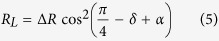


Therefore, in the intermediate state *R*_*I*_ can be expressed as Eq. (6).


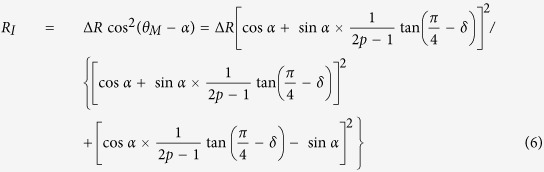


where 
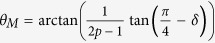
, 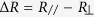, *α* = 6.3° and *δ* = 4.5°. The values of *p* vs *θ*_*H*_ can be plotted in Fig. 4(a) according to Fig. 3(c) and Eq. (6).

On the other hand, the direction of *M* is also involved in the magnetic total energy *E*. The switching of *M* cross the 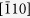 axis must overcome the energy between minima at 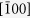 and [010]^24^. This energy is then given by Eq. (2):


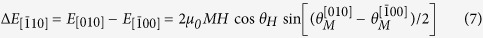



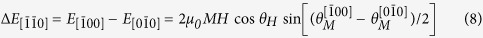


where 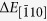 is the energy difference between [010] and 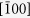 transition; 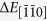 is the energy difference between 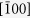 and 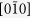 transition. Since the energy difference Δ*E* varies as 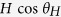 in Eqs. (7) and (8), it can be swept continuously by varying *θ*_*H*_. This provides a direct handle for investigating the domain wall pinning energy distribution^10^. As the probed region in figure breaks up into two regions with the different components of *M*, the value of AMR can reflect the fractional areas corresponding to these two components in Fig. 4(a).

On basis of Eqs. (7) and (8), we can get the energy difference Δ*E* by varying *θ*_*H*_. From Fig. 4(a) (p vs *θ*_*H*_) and Eq. (7) (Δ*E* vs *θ*_*H*_), the relationship between fraction *p* and Δ*E* is shown in the inset of Fig. 4(b). The black and red lines represents the switching from *M*_[010]_ to 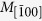 and from 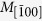 to 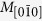, respectively. The distributions of pinning fields were obtained by derivative of *p* with respect to Δ*E/M*_*s*_ as shown in Fig. 4(b), which can be fitted by a Gaussian function^24^. The domain wall pinning fields of 3.78 *Oe* and 4.5 *Oe* were obtained at 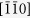 and 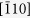, respectively. The difference in domain wall pinning fields at these two axes is related to the superimposed UMA along the 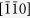 direction, which reduces the energy barrier for this direction.

From the analysis above, the magnetization switching process obeys coherent rotation model at high fields and domain nucleation and propagation at low fields. We investigate the AMR data at the different fields according to those two models in Fig. 3. At high field of 387 *Oe*, only coherent rotation model is used to calculate the AMR, which shows good agreement with experiment as shown in Fig. 3(a). At low field of 5 *Oe*, the four stable plateau of AMR indicates that the domains nucleation and propagation dominates, and the fitting results by domain wall pinning energy are in agreement with the experimental values. When applied magnetic field is between the low field (5 *Oe*) and saturation field, the magnetization switching process involves the domain nucleation and propagation and part of coherent rotation. Due to slight deviation current, coherent rotation of magnetization at easy axis and propagation of domains between two easy axes are observed, which reflects in the tilted plateau and jump of AMR. The domain wall pinning and coherent rotation analysis as above were used to the fitting data at unsaturated fields. For *H* = 15 *Oe* as an example, the coherent rotation is dominated firstly. When the applied field rotates to a certain angle (103.5°) and energy differences between neighboring minima is larger than domain wall pinning energy, the near 90° domain switching appears. The *K*_*U*_ and *K*_1_ obtained at high field and domain wall pinning field obtained at low field are used to fit the AMR of 15 *Oe*, which is the blue and green solid line shown in Fig. 3(b).

**Figure 5:**
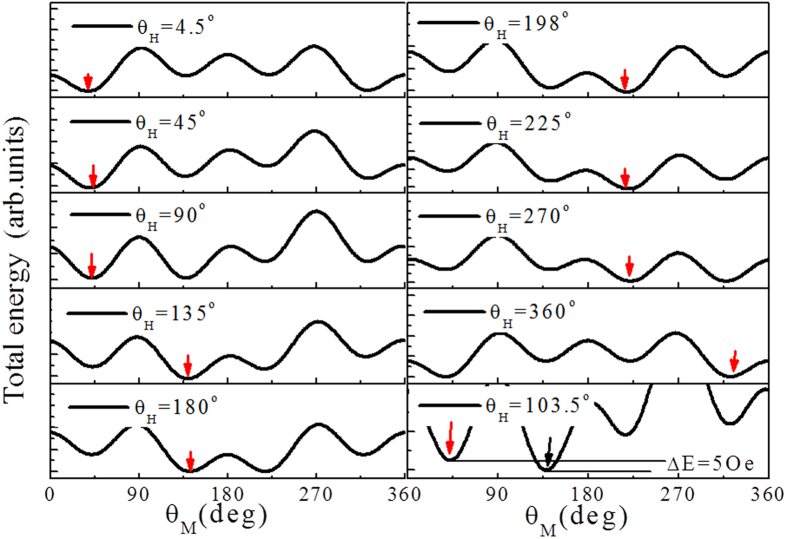
The evolution of magnetic total energy density with rotating field direction such as for H = 15 *Oe*. The solid red rows at the minima indicate the orientations of magnetization. Following the direction of the magnetic field rotating 360°, the direction of magnetic domains in the sample follows 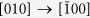 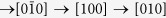 reorientation.

All the behavior of AMR can be explained by total energy density *E* given in Eq. (2), which includes four-fold anisotropy energy, UMA energy, and Zeeman energy. Figure 5 shows the variation of *E* at different direction of the fixed field *H* = 15 *Oe*. The four minima positions can be clearly observed. The solid red rows at the minima indicate the orientations of magnetization. The first panel at *θ*_*H*_ = 4.5° indicates that the entire sample is magnetized along a near Fe[010] direction. Following the direction of the magnetic field rotating 360°, the direction of magnetization in the sample follows 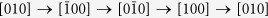 orientation as shown in Fig. 5.

In contrast to the coherent rotation model, which means that the magnetization locates at the position of total energy minima, the magnetization position is actually not at energy minima in some values *θ*_*H*_ of unsaturation magnetic field. For *H* = 15 *Oe*, when *θ*_*H*_ = 103.5°, the direction of magnetization is still at Fe[010] axis, but the minimum energy is at 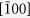 direction. Therefore, the energy difference between two directions is domain wall pinning energy. Interestingly, the value of the domain wall pinning field governed the switching of Fe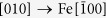, 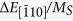 = 5 *Oe*, is comparable with the experimental results.

## Discussion

The magnetization switching process in Fe/MgO(001) film, which is dominated by both magnetic anisotropy energy and domain wall pinning energy, was investigated by AMR technique. In order to deduce magnetic anisotropy constants and domain wall pinning energy, the current is applied at an angle of 6.3° with respect to the hard axis (Fe[110]) direction) for AMR measurements. This configuration can distinguish the magnetization along the two easy axes and detect the small rotation of magnetization in easy axis direction. The AMR curves at magnetic fields high enough to ensure the coherent rotation of magnetization indicate a smooth behavior without hysteresis between CW and CCW rotations. By analyzing magnetic torque, the values and orientations of *K*_*U*_ and *K*_*1*_ can be confirmed. On the other hand, the AMR curves at low fields show abrupt transitions with hysteresis between CW and CCW rotations, suggesting the presence of multi-domain structures in the abrupt transition regions. When the applied field is far small than unsaturated field (~5 *Oe*), the domain wall pinning energy is obtained by analysis different behavior of AMR.

## Methods

The Fe/MgO(001) film was prepared by molecular-beam epitaxy (MBE) in an ultrahigh vacuum (UHV) system with a base pressure of 2.0 × 10^−10^ *mbar*. After transferred into the UHV chamber, the MgO (001) substrate was first annealed at 700 °C for 2 hours to obtain clean surfaces. Fe film with thickness of 4.2 *nm* was grown at room temperature with a deposition rate 0.2 *nm/min*. Moreover, 4.5 *nm* Cu film was deposited on the Fe film as a capping layer to prevent sample from oxidization. The magneto-optical Kerr effect (MOKE) measurement was performed to confirm the magnetic properties. The angular dependence AMR measurements with a standard four-point method were carried out at room temperature and the details are described in ref. 14. The measuring time of one AMR point is far larger than magnetization switching time, and consequently the magnetization is always in equilibrium state during measurement. The current is applied at an angle of 6.3° with respect to the Fe[110]) direction to distinguish two components of magnetizations along the two easy axes.

## Additional Information

**How to cite this article**: Hu, B. *et al.* Determination of magnetic anisotropy constants and domain wall pinning energy of Fe/MgO(001) ultrathin film by anisotropic magnetoresistance. *Sci. Rep.* **5**, 14114; doi: 10.1038/srep14114 (2015).
